# Effectiveness of internet-based interventions for children, youth, and young adults with anxiety and/or depression: a systematic review and meta-analysis

**DOI:** 10.1186/1472-6963-14-313

**Published:** 2014-07-18

**Authors:** Xibiao Ye, Sunita Bayyavarapu Bapuji, Shannon Elizabeth Winters, Ashley Struthers, Melissa Raynard, Colleen Metge, Sara Adi Kreindler, Catherine Joan Charette, Jacqueline Angela Lemaire, Margaret Synyshyn, Karen Sutherland

**Affiliations:** 1 Centre for Healthcare Innovation Evaluation Platform, Winnipeg Regional Health Authority, 200-1155 Concordia Avenue, Winnipeg, Manitoba R2K 2M9, Canada; 2 Department of Community Health Sciences, Faculty of Medicine, University of Manitoba, Winnipeg, Manitoba, Canada; 3 Concordia Hospital Library, University of Manitoba, 1095 Concordia Avenue, Winnipeg, Manitoba R2N 3S8, Canada; 4 Addictions Foundation of Manitoba, 1031 Portage Avenue, Winnipeg, Manitoba R3G 0R8, Canada; 5 Manitoba Adolescent Treatment Centre, 120 Tecumseh St, Winnipeg, Manitoba R3E 2A9, Canada; 6 Health and Rehabilitation Sciences, Faculty of Health Sciences, Western University, London, ON N6G 1H1, Canada; 7 Mental Health Crisis Response Centre, Winnipeg Regional Health Authority, 817 Bannatyne Avenue, Winnipeg MB R3E 0W4, Canada

**Keywords:** Internet-based intervention, Anxiety, Depression, Child and youth, Effectiveness

## Abstract

**Background:**

The majority of internet-based anxiety and depression intervention studies have targeted adults. An increasing number of studies of children, youth, and young adults have been conducted, but the evidence on effectiveness has not been synthesized. The objective of this research is to systematically review the most recent findings in this area and calculate overall (pooled) effect estimates of internet-based anxiety and/or depression interventions.

**Methods:**

We searched five literature databases (PubMed, EMBASE, CINAHL, PsychInfo, and Google Scholar) for studies published between January 1990 and December 2012. We included studies evaluating the effectiveness of internet-based interventions for children, youth, and young adults (age <25 years) with anxiety and/or depression and their parents. Two reviewers independently assessed the risk of bias regarding selection bias, allocation bias, confounding bias, blinding, data collection, and withdrawals/dropouts. We included studies rated as high or moderate quality according to the risk of bias assessment. We conducted meta-analyses using the random effects model. We calculated standardized mean difference and its 95% confidence interval (95% CI) for anxiety and depression symptom severity scores by comparing internet-based intervention vs. waitlist control and internet-based intervention vs. face-to-face intervention. We also calculated pooled remission rate ratio and 95% CI.

**Results:**

We included seven studies involving 569 participants aged between 7 and 25 years. Meta-analysis suggested that, compared to waitlist control, internet-based interventions were able to reduce anxiety symptom severity (standardized mean difference and 95% CI = −0.52 [−0.90, −0.14]) and increase remission rate (pooled remission rate ratio and 95% CI =3.63 [1.59, 8.27]). The effect in reducing depression symptom severity was not statistically significant (standardized mean difference and 95% CI = −0.16 [−0.44, 0.12]). We found no statistical difference in anxiety or depression symptoms between internet-based intervention and face-to-face intervention (or usual care).

**Conclusions:**

The present analysis indicated that internet-based interventions were effective in reducing anxiety symptoms and increasing remission rate, but not effective in reducing depression symptom severity. Due to the small number of higher quality studies, more attention to this area of research is encouraged.

**Trial registration:**

PROSPERO registration: CRD42012002100

## Background

Up to 20% of children, youth, and young adults are affected by mental disorders each year [1,2], but less than 50% of those patients received specialized treatment services [2,3]. Modern information technologies offer new opportunities to deliver mental health interventions via computer-based or mobile phone based internet. The majority of internet-based mental health interventions have been aimed at adults [4], particularly those with anxiety and depression disorders [5-10]. These studies have shown that internet-based interventions were feasible and improved access and patient mental health outcomes in adults.

With the dramatically increased adoption of internet-based devices among the young population, studies have recently started to include children, youth, and young adults with anxiety and depression concerns [11-16]. Two recent narrative reviews have overviewed the findings of internet-based programs for anxiety and depression in children, youth, and young adults [17,18], but neither of them calculated pooled effect estimates using meta-analysis methods. Internet-based mental health services may lower the overall cost by saving staff and client time and by minimizing the use of other resources such as clinic rooms [19,20], but the empirical evidence has not been systematically examined. We sought to systematically review the most recent findings and calculate overall (pooled) effect estimates of internet-based anxiety and depression interventions in children, youth and young adults.

## Methods

Detailed analysis protocol was registered with the International Prospective Register of Systematic Reviews (PROSPERO registration number: CRD42012002100).

### Inclusion and exclusion criteria

We used the P.I.C.O.S. (Population, Interventions, Comparators, Outcomes, and Study Design) framework to identify relevant studies. Our focus was on children, youth, and young adults (age <25 years). Studies targeting parents of children (especially young children) with a mental health disorder/problem were also included. We excluded studies that did not clearly state study population characteristics. Interventions of interest were those targeting anxiety and/or depression symptoms and were delivered via the Internet (fixed or mobile internet). We considered both parallel comparisons (randomized or non-randomized controlled trials), pre-/post-intervention comparison studies, and observational studies. The primary outcomes of interest were anxiety and/or depression symptom severity and diagnosis (e.g., symptom scale scores and remission rates). We excluded studies solely evaluating participant expectations, experiences, and/or acceptance.

### Literature search

A health science librarian (M.R.) conducted a literature search of multiple bibliographic databases including PubMed/Medline, EMBASE, CINAHL, PsychInfo/Proquest and Google Scholar (1990–2012) using both subject headings/terms and free text keywords. Heading and free text terms used to capture the concept of electronic provision of services included: e-mental, emental, ehealth, e-health, internet, virtual, mobile health, mhealth, m-health, mobile phone, cell phone, cellular phone, smartphone, iphone, tablet, ipad, information communication technology, text message, mobile message, message boards, social media, facebook, twitter, myspace, google+, blogging, and telemedicine. These terms were then combined with subject and free text terms describing mental health services (e.g., delivery of health care, health services accessibility, delivery of mental health services, mental health services, community mental health services, etc.) and terms describing youth, children and adolescents to capture the literature containing information about the electronic provision of mental health services to this age group. This search strategy was designed for PubMed/Medline, then translated for use in the other databases. A Google search using the search terms mentioned above was also undertaken to locate grey literature. We limited our search to articles published since 1990.

Titles and abstracts of articles were scanned by two reviewers (S.B.B. and S.E.W.) independently to make an initial assessment of relevance based on the inclusion and exclusion criteria. The two reviewers met regularly to reach a consensus on relevance of each title and abstract. When the information was not enough to judge the relevance, the full-text was retrieved. Articles that did not provide sufficient information on the P.I.C.O.S. framework elements or did not meet the inclusion criteria as defined using the framework were considered irrelevant and were thus excluded. Reviewers also hand-searched references cited in those articles to maximize the number of studies identified.

### Study appraisal and selection

Two reviewers (S.B.B. and S.E.W.) independently evaluated the quality of screened studies using a modified version of the Quality Assessment Tool for Quantitative Studies [21] and rated each of the six quality components: selection bias (bias caused by systematic differences between those who are selected for a study and those who are not), allocation bias (bias caused by non-random allocation of participants to intervention and control groups), confounding bias (bias due to the presence of a common cause of exposure/intervention and outcome), blinding (researchers and/or participants are unaware of the group to which the participants are assigned to), data collection methods (validity and reliability of data collection tools), and withdrawals/dropouts (the percentage of participants that do not complete the study or dropped out). Reviewers independently rated each component as strong, moderate, or weak and assigned a global quality rating to each study: strong quality (four strong ratings with no weak ratings); moderate quality (less than four strong ratings and one weak rating); weak quality (two or more weak ratings). Reviewers met regularly to reach a consensus and disagreements were discussed and resolved in team meetings with senior investigators (X.Y. and C.M.). We developed a data extraction form and pilot-tested it on five articles. A reviewer extracted information on study characteristics (e.g., design, location), participant characteristics (e.g., age, sex, enrollment approach), intervention (e.g., number of intervention groups, comparison, intervention details), and results. A second reviewer checked the extracted data and any disagreements were solved between the two reviewers or by involving a third author when necessary.

### Data analysis

We included high or moderate quality studies in the meta-analysis. We used the random effects meta-analysis model to estimate pooled intervention effects. Effect estimates based on intent-to-treat analysis were used whenever applicable; otherwise complete-sample-analysis results were used. We examined heterogeneity among studies using Cochran’s Q test and Higgins’ I^2^ statistics according to the Cochrane Handbook [22]. I^2^ < 30% and I^2^ = 30-50% were considered the presence of minimal heterogeneity and moderate heterogeneity, respectively [22]. For continuous outcomes (i.e., anxiety/depression symptom scores), we calculated standardized mean differences and its 95% confidence intervals (95% CIs) between an internet-based intervention and control and between an internet-based intervention and face-to-face intervention (or usual care) [22]. For the binary outcome (i.e., remission rate), we calculated a pooled rate ratio and 95% CI. To test the robustness of the pooled effect estimates, we reran the models by using anxiety/depression outcomes measured by alternative instruments (each study assessed anxiety/depression symptoms using multiple instruments simultaneously). We also reran the model after excluding studies that were not CBT based. We undertook a subgroup analysis by methodology quality rating (strong vs. moderate). Significant level was set at 0.05. We rated the quality of the synthesized evidence using the GRADE approach [23]. All analyses were undertaken in RevMan 5.2.

## Results

**Figure 1:**
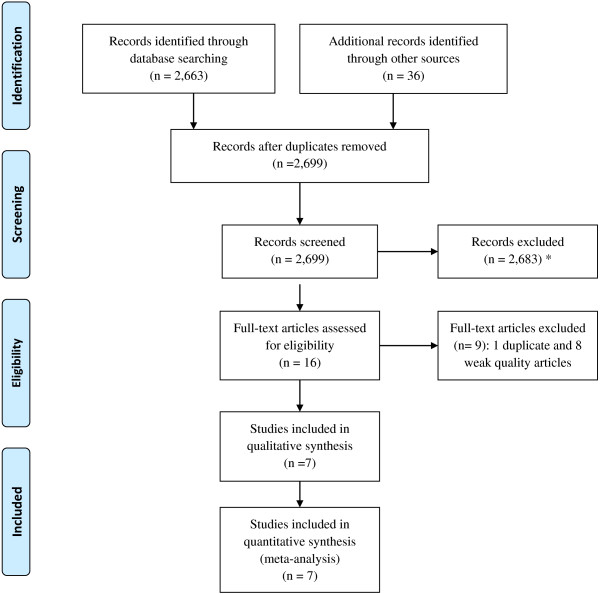
**Study selection and exclusion flow diagram.** Identification, number of articles identified through the literature search including grey literature; Screening, number of articles screened according to the criteria described in main text; Eligibility: number of screened articles that met the inclusion criteria; Included, number of studies included in the review and meta-analysis. *Literature search identified studies on other mental health issues as a part of the research project but those studies were not included in the present analysis.

Figure 1 describes the process and the number of studies reviewed at each step. We included three strong and four moderate quality studies, all randomized controlled trials (RCTs), in this analysis after excluding eight weak quality studies and one partially duplicate study (see Additional file 1: Table S1). Of the seven studies, six were published in peer-reviewed journals, and one was a doctoral dissertation [11]. These studies enrolled a total of 569 participants aged between 7 and 25 years (Table 1). Participants were diagnosed with anxiety disorders in four studies, with anxiety and/or depression in one study, and did not have a specified diagnosis in two studies (but focusing on reducing participants’ anxiety and depression symptoms). All studies but one [13] explicitly stated that cognitive behavioral therapy (CBT) was applied in the interventions. Intervention group participants in this study used a mobile phone based tool to self-monitor their mood, stress, and alcohol and cannabis use daily and received short text message (SMS) and phone call supports from psychologists [13]. Interventions in all studies included online self-help sessions; six studies supplemented online self-help with therapist support via email, SMS, and/or phone call (one of the six studies also included family support [16]); and one study included school-based group support and teacher support [12]. Participants accessed to the online intervention contents at home or school (the same settings where they normally access the internet). The duration of interventions ranged from 3 to 12 weeks. All included studies compared an internet-based intervention to a waitlist control group and two also compared an internet-based intervention to a face-to-face intervention (or usual care). Six studies measured both anxiety and depression symptoms as primary outcomes and the remaining one focused on depression symptoms only [12]. All studies used more than one outcome instrument, but no single instrument was used in all of the studies. Outcomes were measured pre- and post-intervention at different time points (up to 12 months).

**Table 1:** Characteristics of Studies of Internet-based anxiety and depression interventions among children, youth, and young adults

**Reference**	**Study design**	**Participant**	**No. of participants**	**Intervention**		**Outcome**	
				**Intervention contents**	**Intervention duration**	**Primary outcomes**	**Time of measurement**
Keller, 2010 [11]	2-arm RCT (Internet program vs. Waitlist control)	Children with anxiety and mothers	37	Computer-based CBT program (self-help + therapist support)	12 weeks	Anxiety, depression, and social phobia symptom assessment scores	Baseline, 6 and 12 weeks after the intervention
March, 2009 [24]	2-arm RCT (Internet-based CBT vs. Waitlist control)	Children (7–12 years) with anxiety disorders and parents	73	Computer -based CBT program (self-help sessions + therapist support through email and phone)	10 weeks for children and 6 weeks for parents (60 minutes per session)	Anxiety diagnostic status and severity, number of anxiety diagnoses, anxiety and depression symptom assessment scores	Baseline, the end of intervention, and 6 months after the intervention (for intervention group only)
Storch, 2011 [16]	2-arm RCT (Internet-based CBT vs. Waitlist control)	Children and adolescents (7–16 years) with obsessive compulsive disorder and at least one parent	31	Family based CBT treatment delivered via computer-based internet (self-help sessions + online therapist support)	12 weeks	Anxiety and depression symptom assessment scores, remission rate post intervention	Baseline and the end of intervention
Spence, 2011 [14]	3-arm RCT (Internet-based CBT vs. Clinic-based CBT vs. Waitlist control)	Adolescents (12–18 years) with anxiety disorders and parents	115	Computer -based CBT treatment (self-help session + online therapist support through email) or clinic-based CBT treatment	10 weeks	Anxiety diagnostic status, anxiety and depression symptom severity	Baseline, 3, 6, and 12 months after the intervention
O’Kearney, 2009 [12]	2-arm RCT (Internet-based curriculum vs. Waitlist control)	Adolescent girls (15–16 years)	157	Computer -based CBT program MoodGYM (self-help + school-based group support + teacher support)	6 weeks (for 3 modules)	Depression diagnosis and symptom assessment scores, attributional style	Baseline, the end of intervention, and 20 weeks after the intervention
Sethi, 2010 [15]	4-arm RCT (Internet-based CBT vs. Face-to-face CBT vs. Combined face-to-face/online CBT vs. Control)	Students (15–25 years) with low or moderate level of anxiety/depression	38	Computer -based CBT program MoodGYM delivered at school or at home (self-help sessions + therapist support)	5 sessions within 3 weeks	Depression, anxiety, and distress symptoms	Baseline and the end of intervention
Reid, 2011 [13]	2-arm RCT (Mobile phone-based intervention vs. Control)	Youth with mild or more severe emotional/mental health issue but no severe psychiatric condition	118	Mobile phone-based self-monitoring + SMS and phone call support by psychologist	6 weeks	Depression, anxiety, and distress symptoms	Baseline, the end of intervention, and 6 weeks after the intervention

CBT, cognitive behavioral therapy; RCT, randomized controlled trial; SMS, short text message.

**Figure 2:**
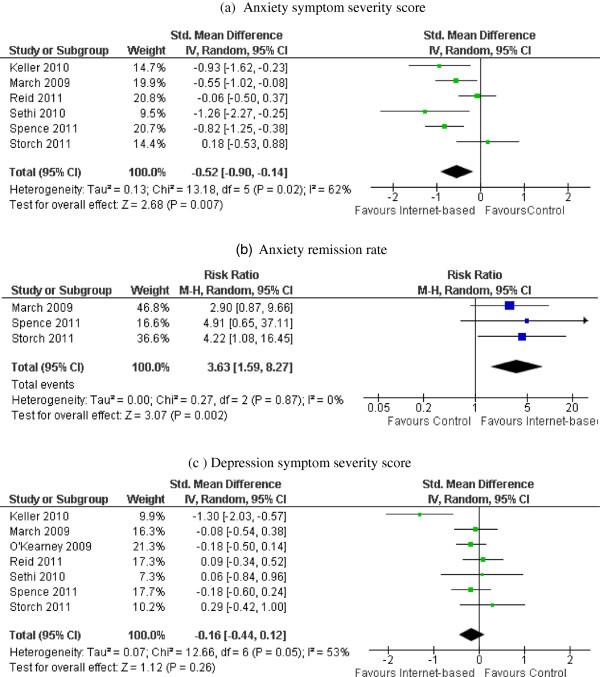
**Post-intervention anxiety/depression outcomes: internet-based intervention vs. waitlist control.** Forest plot of standardized mean differences/risk ratio (squares, proportional to weights used in meta-analysis) and associated confidence intervals (lines). Summary measure and 95% confidence interval is presented as a diamond. Panel **a**: Forest plot of standardized mean differences in anxiety symptom severity score. Panel **b**: Forest plot of relative risk for anxiety symptom remission. Panel **c**: Forest plot of standardized mean differences in depression symptom severity score. CI, confidence interval; df, degrees of freedom; Chi^2^, statistical test for heterogeneity; P, p-value of Chi^2^ (evidence of heterogeneity of intervention effects); I^2^, amount of heterogeneity between trials; Z, test for overall effect; Overall effect P, p-value for significance of overall effect.

We first compared internet-based interventions to waitlist control. Meta-analysis suggested that internet-based interventions were able to reduce anxiety symptom severity compared to waitlist control (standardized mean difference and 95% CI = −0.52 [−0.90, −0.14], p for heterogeneity test = 0.02, I^2^ = 62%), as shown in Figure 2(a). Sensitivity analysis, by removing the study that was not described as CBT-based, did not change the overall effect estimate. Participants receiving internet-based interventions were also more likely than waitlist controls to be free of a diagnosis of anxiety disorder after the treatment (remission rate ratio and 95% CI = 3.63 [1.59, 8.27], p for heterogeneity test = 0.87, I^2^ = 0%), Figure 2(b). However, the effect in reducing depression symptom severity was not statistically significant (standardized mean difference and 95% CI = −0.16 [−0.44, 0.12], p for heterogeneity test = 0.05, I^2^ = 53%), Figure 2(c). Subgroup analysis has shown similar results between studies with different quality ratings (strong vs. moderate).

**Figure 3:**
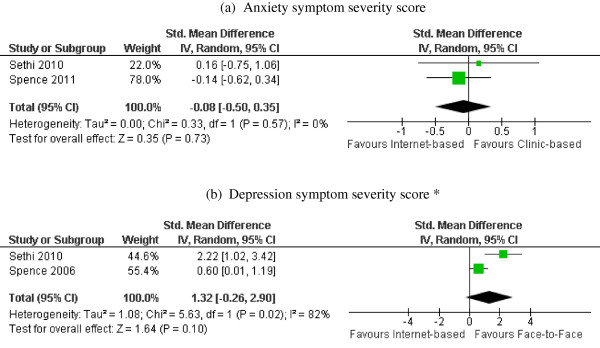
**Post-intervention anxiety/depression symptom scores: internet-based intervention vs. face-to-face intervention.** Forest plot of standardized mean differences (squares, proportional to weights used in meta-analysis) and associated confidence intervals (lines). Summary measure and 95% confidence interval is presented as a diamond. Panel **a**: Forest plot of standardized mean differences in anxiety symptom severity score. Panel **b**: Forest plot standardized mean differences in depression symptom severity score. (*Depression data were reported in (S. H. Spence, Holmes, March, & Lipp, 2006), an earlier analysis of the study (S. H. Spence et al., 2011)).

The meta-analysis of the two studies comparing internet-based intervention to face-to-face intervention showed no statistical differences in intervention effects of anxiety symptoms (standardized mean difference and 95% CI = −0.08 [−0.50, 0.35], p for heterogeneity test = 0.57, I^2^ = 0%) or depression symptoms (standardized mean difference and 95% CI = 1.32 [−0.26, 2.90], p for heterogeneity test = 0.02, I^2^ = 82%), as shown in Figures 3(a) and (b).

The majority of studies examined short term effects (effects at the end of the intervention or less than 12 weeks post intervention). Two studies followed the intervention group participants but not the control group participants 6 months and 12 months after the interventions, respectively, and found the improvement (compared to pre-intervention) retained [14,24]. However, only one study followed participants in both the intervention and control groups for 12 months after the intervention [14] and showed no intervention effect at this time point. In one study [11], participants had a lower anxiety level 6 weeks after the intervention, but this effect disappeared 12 weeks after the intervention.

## Discussion

Our analyses demonstrated that internet-based interventions were effective in reducing anxiety symptom severity compared to no intervention, and this effect may be equal to that of face-to-face interventions (or usual care). The findings support the observations in the two previous narrative reviews where the majority of studies showed positive effects [17,18]. This is also consistent with the findings from meta-analyses of internet-based interventions in adults [9,25,26]. However, the quality of the evidence was low to moderate (see Additional file 1: Table S2). The limited extant findings suggest that augmentation of the internet interventions are required to maintain the positive effects.

The analysis, however, did not support the effect of internet-based interventions on reducing depression symptoms. The difference in the effects on the two disorders is consistent with findings from one previous systematic review of internet-based interventions for adult depression and anxiety [25]. The meta-analysis found a larger effect size for anxiety than for depression, which the authors believed was explained by the magnitude of therapist involvement but not the type of disorder. Therapist involvement might also explain the difference found in the present analysis since the study focusing on depression only was the only one without therapist support (although school and family supports were provided) [12]. Furthermore, more than half of the interventions in the present analysis were developed to primarily target anxiety disorders. Despite the high prevalence of comorbidity in the young population, depression and anxiety disorders are two different disorders with their own behavioral symptoms [27]. CBT interventions for the two disorders often contain similar contents [28]. Transdiagnostic CBT, an approach bringing therapeutic elements from disorder-specific CBTs together to treat diagnostically mixed patients, may offer several advantages but there is no sufficient evidence supporting the effectiveness of this approach versus control [29]. Few studies have found that internet-based transdiagnostic CBT improved patient outcomes when compared to control [30]. However, it is unclear whether transdiagnostic CBT performs better than disorder-specific CBTs [31]. Previous studies have indicated that even if a CBT intervention was effective in reducing anxiety or depression symptoms, the intervention might not work for the other comorbid condition [28]. Therefore, more research is needed to compare internet-based transdiagnostic CBT vs. disorder-specific CBT for anxiety and depression.

The interpretation of the findings from the current analysis needs to consider several factors. First, the combined effect estimates do not take into account baseline differences between comparative groups. Some of the included studies have found statistical differences in demographic characteristics and/or baseline anxiety/depression symptom scores between the participants in the intervention group and those in the control group [12,14,16]. Methods including change-from-baseline comparison, covariance analysis, and regression models can be used to adjust for baseline differences, but there were not enough data from the included studies to conduct a meta-analysis. Second, studies included in this analysis involved patients with mild or moderate anxiety/depression symptoms [13-16]. While the analysis indicates that internet-based intervention can improve symptoms in those patients, the interventions may not affect patients with severer symptoms. Third, intervention duration and outcome follow-up length varied across studies. We were not able to examine the long-term effect (greater than 12 weeks after the intervention), but individual studies have indicated that the intervention effect might not last over a long period of time [11,14]. Future studies should follow participants from both intervention and control groups for a longer time period in order to examine effect maintenance.

Many RCTs (particularly mobile device-based studies) initiated recently are still recruiting participants [32-34], therefore, were not included in the current analysis. The adoption of mobile devices (e.g., cell phone, smartphone, and tablet) has been dramatically increasing among the young population. Almost 80% of teens in America have a cell phone and almost half of them own smartphones [35]. Around a quarter of them also have a tablet computer [35]. Mobile devices offer greater mobility and provide new opportunities to enhance the delivery of mental health and other medical services [36,37]. With only one mobile phone-based study in the present analysis, we were not able to compare the effects of fixed internet-based interventions versus mobile-based interventions. A recent systematic review [38] found mobile phone based diabetes self-management had a larger effect than computer-based intervention. There was, however, no difference in effect size between mobile phone-based and computer-based adult depression interventions [39]. Given the rapid growth of mobile phone users (in particular smartphone users) and the advantages of mobile technologies, more studies are needed to examine the effectiveness of mobile phone-based intervention.

None of the included studies in this analysis evaluated the cost or the cost-effectiveness of internet-based interventions for children. Adult studies suggest computerized or internet-based CBTs for anxiety and depression are cost-effective [7,40,41]. These findings may not apply to children and youth because interventions for these patients usually require parent and school teacher involvement. Future studies should collect comprehensive cost data and long-term effect data in order to conduct economic analysis.

There are methodological limitations in the present meta-analysis. We included studies published in English only. This analysis was based on a small number of studies with inconsistent approaches for data analysis (i.e., complete-sample-analysis vs. intent-to-treat analysis). Each study used several different instruments to assess anxiety and/or depression symptoms but only those measurements from instruments that were used more commonly across the studies (e.g., Beck Anxiety Inventory and Children’s Depression Inventory) were included in the meta-analyses. However, replacing the outcomes with those from less commonly used instruments did not significantly change the combined effect estimates.

## Conclusions

In conclusion, the analysis indicated that internet-based interventions were effective in reducing anxiety symptoms, and might be as effective as face-to-face interventions. However, the interventions may not work for depression. Stronger evidence is needed and future studies should also examine whether or not the interventions are cost-effective. Given the rapid adoption of mobile devices among children, youth, and young adults, it is also important to develop and evaluate mobile device based interventions.

## Abbreviations

CBT: Cognitive behavioral therapy; P.I.C.O.S.: Population, intervention, comparator, outcome, and study design; 95% CI: 95% confidence interval; RCTs: Randomized controlled trials.

## Competing interests

No disclosed financial or nonfinancial competing interests by all authors.

## Authors’ contributions

XY conceived of the study, and led the design and execution of the synthesis and drafted the manuscript. CM and MS, a nominated principal applicant and a principal knowledge user, participated in designing the study and supervised the conduction of the study. SB and SW independently screened and assessed the quality of studies. MR conducted systematic literature search. AS, JL, CC, SK, and KS participated in quality assessment, data analysis and interpretation. All authors read and approved the final manuscript.

## Pre-publication history

The pre-publication history for this paper can be accessed here:

http://www.biomedcentral.com/1472-6963/14/313/prepub

## Supplementary Material

Additional file 1: Table S1 Quality assessment of included studies. **Table S2.** Summary of Findings and Quality of Evidence. Click here for file
