# Supplementary material for: Effectiveness of internet-based interventions for children, youth, and young adults with anxiety and/or depression: a systematic review and meta-analysis
Source: BMC Health Serv Res. 2014 Jul 18;14:313. doi: 10.1186/1472-6963-14-313 (PMC4110069; doi:10.1186/1472-6963-14-313)
Supplement: Additional file 1: Table S1 — Quality assessment of included studies. Table S2. Summary of Findings and Quality of Evidence. [file 1472-6963-14-313-S1.doc]

**Additional file 1: Table S1** Quality assessment of included studies

| Reference | Selection bias | Allocation bias | Adequate adjustment for confounders | Blinding | Validity and reliability of data collection methods | Withdraw and drop-outs | Global rating |
| --- | --- | --- | --- | --- | --- | --- | --- |
| Keller, 2010 [11] | Moderate | Strong | Moderate | Moderate | Strong | Strong | Moderate |
| March, 2009 [24] | Strong | Strong | Strong | Moderate | Strong | Strong | Strong |
| Storch, 2011 [16] | Moderate | Strong | Strong | Moderate | Strong | Strong | Strong |
| Spence, 2011 [14] | Moderate | Strong | Strong | Moderate | Strong | Strong | Strong |
| O’Kearney, 2009 [12] | Weak | Moderate | Strong | Moderate | Strong | Moderate | Moderate |
| Sethi, 2010 [15] | Moderate | Strong | Weak | Moderate | Strong | Strong | Moderate |
| Reid, 2011 [13] | Strong | Strong | Strong | Moderate | Strong | Strong | Strong |

**Additional file 1 Table S2 Summary of Findings and Quality of Evidence**

| **Anxiety and depression symptom severity scores** | | | | | | |
| --- | --- | --- | --- | --- | --- | --- |
| **Patient or population:** patients with anxiety and/or depression **Settings:** interventions delivered via the Internet **Intervention:** internet-based behavioral interventions **Comparison:** waitlist control | | | | | | |
| **Outcomes** | **Illustrative comparative risks* (95% CI)** | | **Relative effect (95% CI)** | **No of Participants (studies)** | **Quality of the evidence (GRADE)** | **Comments** |
|  | Assumed risk | Corresponding risk |  |  |  |  |
|  | **Waitlist control** | **Internet-based behavioral interventions** |  |  |  |  |
| **Anxiety symptom score** Internet-based *vs.* control | The standardized mean symptom scores ranged across control groups from -1.26 to 0.18 | The standardized mean anxiety symptom score in the intervention groups was **0.52 standard deviations lower** (0.9 to 0.14 lower) |  | 333 (6 studies) | ⊕⊕⊕⊝ **moderate**^1^ | Lower score indicated improvement |
| **Depression symptom score** Internet-based *vs.* control | The standardized mean symptom scores ranged across control groups from -1.30 to 0.29 | The standardized mean depression symptom score in the intervention groups was **0.16 standard deviations lower** (0.44 lower to 0.12 higher) |  | 490 (7 studies) | ⊕⊕⊝⊝ **low**^1,2^ | Lower score indicated improvement |
| **Anxiety symptom score** Internet-based *vs.* face-to-face | The standardized mean symptom scores ranged across control groups from -0.14 to 0.16 | The standardized mean anxiety symptom score in the intervention groups was **0.08 standard deviations lower** (0.5 lower to 0.35 higher) |  | 90 (2 studies) | ⊕⊕⊕⊝ **moderate**^3^ |  |
| **Depression symptom score** Internet-based *vs.* face-to-face | The standardized mean symptom scores ranged across control groups from 0.60 to 2.21 | The standardized mean depression symptom score in the intervention groups was **1.32 standard deviations higher** (0.26 lower to 2.9 higher) |  | 66 (2 studies) | ⊕⊕⊕⊝ **moderate**^3^ |  |
| **Remission rate**  (end of intervention) | **Moderate**^4^ | | **RR 3.63**  (1.59 to 8.27) | 161 (3 studies) | ⊕⊕⊕⊝ **moderate** |  |
|  | **103 per 1000** | **374 per 1000** (164 to 852) |  |  |  |  |
| *The basis for the **assumed risk** (e.g. the median control group risk across studies) is provided in footnotes. The **corresponding risk** (and its 95% confidence interval) is based on the assumed risk in the comparison group and the **relative effect** of the intervention (and its 95% CI). **CI:** Confidence interval; **SMD**: standardized mean difference; **RR:** Risk ratio; | | | | | | |
| GRADE Working Group grades of evidence **High quality:** Further research is very unlikely to change our confidence in the estimate of effect.  **Moderate quality:** Further research is likely to have an important impact on our confidence in the estimate of effect and may change the estimate. **Low quality:** Further research is very likely to have an important impact on our confidence in the estimate of effect and is likely to change the estimate. **Very low quality:** We are very uncertain about the estimate. | | | | | | |
| ^1^ Presence of heterogeneity (I-squared=62%, p=0.02) ^2^ One study was ranked weak for selection bias and another study was ranked weak for confounder adjustment ^3^ Inadequate adjustment of confounders  ^4^ The population in control group with moderate risk | | | | | | |
